# Dual Inhibitory Action of a Novel AKR1C3 Inhibitor on Both Full-Length AR and the Variant AR-V7 in Enzalutamide Resistant Metastatic Castration Resistant Prostate Cancer

**DOI:** 10.3390/cancers12082092

**Published:** 2020-07-28

**Authors:** Mona Kafka, Fabian Mayr, Veronika Temml, Gabriele Möller, Jerzy Adamski, Julia Höfer, Stefan Schwaiger, Isabel Heidegger, Barbara Matuszczak, Daniela Schuster, Helmut Klocker, Jasmin Bektic, Hermann Stuppner, Iris E. Eder

**Affiliations:** 1 Department of Urology, Medical University Innsbruck, 6020 Innsbruck, Austria; mona.kafka@tirol-kliniken.at (M.K.); Hoefer_jul@yahoo.de (J.H.); isabel.heidegger@tirol-kliniken.at (I.H.); helmut.klocker@i-med.ac.at (H.K.); jasmin.bektic@tirol-kliniken.at (J.B.); 2 Institute of Pharmacy/Pharmacognosy, Center for Molecular Biosciences Innsbruck (CMBI), University of Innsbruck, 6020 Innsbruck, Austria; f.mayr@uibk.ac.at (F.M.); veronika.temml@uibk.ac.at (V.T.); Stefan.Schwaiger@uibk.ac.at (S.S.); hermann.stuppner@uibk.ac.at (H.S.); 3 Research Unit Molecular Endocrinology and Metabolism, Helmholtz Zentrum München, 85764 Neuherberg, Germany; gabriele.moeller@helmholtz-muenchen.de (G.M.); adamski@helmholtz-muenchen.de (J.A.); 4 Department of Biochemistry, Yong Loo Lin School of Medicine, National University of Singapore, Singapore 637551, Singapore; 5 Lehrstuhl für Experimentelle Genetik, Technische Universität München, 85354 Freising-Weihenstephan, Germany; 6 Institute of Pharmacy/Pharmaceutical Chemistry, Center for Molecular Biosciences Innsbruck (CMBI), University of Innsbruck, 6020 Innsbruck, Austria; barbara.matuszczak@uibk.ac.at (B.M.); daniela.schuster@pmu.ac.at (D.S.); 7 Institute of Pharmacy, Department of Pharmaceutical and Medicinal Chemistry, Paracelsus Medical University, 5020 Salzburg, Austria

**Keywords:** ARK1C3, enzalutamide resistance, prostate cancer, AR-V7, spheroid culture, cancer-associated fibroblasts (CAFs)

## Abstract

The expanded use of second-generation antiandrogens revolutionized the treatment landscape of progressed prostate cancer. However, resistances to these novel drugs are already the next obstacle to be solved. Various previous studies depicted an involvement of the enzyme AKR1C3 in the process of castration resistance as well as in the resistance to 2nd generation antiandrogens like enzalutamide. In our study, we examined the potential of natural AKR1C3 inhibitors in various prostate cancer cell lines and a three-dimensional co-culture spheroid model consisting of cancer cells and cancer-associated fibroblasts (CAFs) mimicking enzalutamide resistant prostate cancer. One of our compounds, named MF-15, expressed strong antineoplastic effects especially in cell culture models with significant enzalutamide resistance. Furthermore, MF-15 exhibited a strong effect on androgen receptor (AR) signaling, including significant inhibition of AR activity, downregulation of androgen-regulated genes, lower prostate specific antigen (PSA) production, and decreased AR and AKR1C3 expression, indicating a bi-functional effect. Even more important, we demonstrated a persisting inhibition of AR activity in the presence of AR-V7 and further showed that MF-15 non-competitively binds within the DNA binding domain of the AR. The data suggest MF-15 as useful drug to overcome enzalutamide resistance.

## 1. Introduction

Because of the strong relevance of androgens in all stages of prostate cancer (PCa), androgen deprivation therapy (ADT) is a mainstay in the treatment of metastatic PCa. However, progression to castration-resistant prostate cancer (CRPC) frequently occurs after 18 to 36 months on average [1,2]. The process of CRPC is connected to various mechanisms, as reactivation of androgen signaling under castrated conditions, gain-of-function mutations within the androgen receptor (AR) gene, increased AR expression [3,4,5], and elevated intratumoral androgen biosynthesis through upregulation of androgen-synthesizing enzymes like aldo-keto reductase family 1 member C3 (AKR1C3) [6]. The development of novel anti-androgens such as abiraterone acetate or enzalutamide significantly improved the management of CRPC. These anti-androgens intervene with the AR signaling pathway via different mechanisms. Abiraterone acetate inhibits androgen biosynthesis by targeting cytochrome P450 17A1 (CYP17A1, also known as 17α hydroxylase/17,20-lyase), which catalyzes the conversion of pregnenolone to dehydroepiandrosterone (DHEA) in the adrenal glands [1,7]. Enzalutamide inhibits androgen signaling through competitive binding to the AR, resulting in inhibiting translocation of the receptor into the nucleus and its binding to the DNA, leading to a reduced expression of AR-regulated target genes [2,8,9]. Both drugs achieved stunning results. Notably, enzalutamide, which has been previously reserved for patients with metastatic CRPC based on the AFFIRM (2012) [10] and the PREVAIL (2014) [2] trials, has recently been released by the FDA to the use in all patients with CRPC—not metastatic and metastatic—based on the results obtained in the PROSPER trial [11]. Unfortunately, resistances also occur with these novel drugs [12]. An intriguing potential resistance mechanism is the occurrence of AR splice variants, the most frequent being AR-V7. These constitutively active splice variants lack the ligand-binding domain (LBD) and therefore become insusceptible to enzalutamide, which—like androgens—bind to the LBD within the AR protein [13,14,15,16]. Hence, there is an urgent need to develop novel agents that are able to suppress both full-length AR (AR-FL) and AR-V7. Besides AR variants, increased AR expression, mutations within the AR gene (F876L) [17], activation of the interleukin-6-stat3-AR axis [14], elevated expression of the glucocorticoid receptor (GR) [18], and aberrant intratumoral androgen synthesis [19] are likely causes of anti-androgen resistance.

A number of different enzymes are involved in the biosynthesis of intratumoral androgens like CYP17A1 and AKR1C3. Various studies including recently published findings by our group associated increased expression of AKR1C3 with CRPC [6,20,21] as well as with resistances to enzalutamide [14,22,23] and abiraterone acetate [24]. This key enzyme in steroid biosynthesis, also known as 17β-hydroxysteroid dehydrogenase type 5 (17β-HSD5, HSD17B5), catalyzes the conversion of weak androgen precursors, Δ4-androstene-3,17-dione and 5α-androstane-3,17-dione into the more potent androgens testosterone and 5α-dihydrotestosterone [6,25]. Since it acts downstream of CYP17A1, targeting AKR1C3 would not cause an accumulation of deoxycorticosterone in the adrenal glands, and therefore, the co-administration of prednisone would not be necessary [25,26]. In fact, various AKR1C3 inhibitors have been developed in the past as reviewed by Penning et al. [1,27,28,29]. Of notice, some of them were found to exhibit dual-inhibitory function on AKR1C3 and the AR [30,31,32,33]. The AR is a ligand-inducible nuclear steroid receptor transcription factor for testosterone and dihydrotestosterone that consists of an N-terminal domain (NTD) (carrying the constitutive activation function region), a DNA binding domain (DBD), a hinge region, and a ligand binding domain (LBD), the latter being the best studied section of the protein [34]. The AR is an especially plastic protein, with the ability to rapidly change between several conformations [8]. In addition, despite the relatively large number of available crystal structures (82 entries are reported in RCSB PDB (Protein Data Bank) for human AR, accessed 03.04.2020), structural information is mainly available on the LBD. As a matter of fact, most AR targeting drugs were designed to directly bind to this domain [34,35]. There have been several efforts in the past to develop novel AR antagonists that bind to the NTD or the DBD. However, these are tricky due to the largely unstructured and unstable nature of the NTD and the high sequence similarity (77–80%) of the DBD with other steroid receptors like GR [13,34,36].

The aim of our study was to investigate the anti-neoplastic effects of three natural AKR1C3 inhibitors, which originated from the plant *Melodorum fruticosum*, in PCa with an emphasis on their efficacy in overwhelming enzalutamide resistance. To this end, we used 22Rv1 cells, which are enzalutamide resistant and express AR-V7 [37] and an enzalutamide-resistant PCa cell line (DuCaP EnzaR) that has been previously established in our lab [38]. In addition, we used a co-culture spheroid model consisting of DuCaP cells grown together with cancer-associated fibroblasts (CAFs) as 3-dimensional spheroids. Previous findings showed that DuCaP cells acquire enzalutamide resistance when co-cultured with CAFs, which is associated with increased expression of AKR1C3 [23]. In addition, we demonstrate a bi-functional effect of MF-15, the most potent inhibitor, on ARK1C3 and AR, as shown by considerable downregulation of androgen-regulated genes, reduced production of prostate specific antigen (PSA), and a decrease in the expression of AKR1C3, full-length AR (AR-FL) and AR-V7. Furthermore, we provide evidence that MF-15 acts on the AR through binding to the DBD as revealed by molecular docking simulations and reporter gene assays. All experiments were conducted in charcoal-stripped serum to simulate conditions with ongoing ADT in the state of castration-resistance, as performed in clinical routine. These data let us conclude that MF-15 represents a novel potential AKR1C3 inhibitor with bi-functional activity on AKR1C3 and AR and may, therefore, be used to overcome enzalutamide-resistance in PCa.

## 2. Results

### 2.1. Identification of Three Natural Chalcones as Potential AKR1C3 Inhibitors

**Figure 1:**
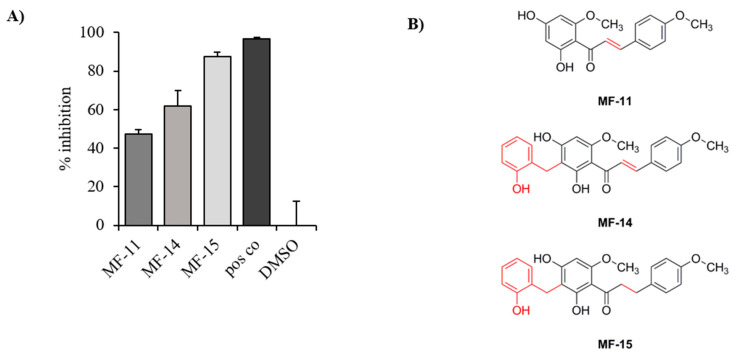
Inhibitory activities towards AKR1C3 of three compounds designated MF-11, MF-14 and MF-15. (**A**) Three compounds with a chalcone and dihydrochalcone scaffold were tested upon their capacity to inhibit AKR1C3 at a concentration of 10 µM using an enzyme inhibition assay as described under material and methods. Data are represented as mean percentage inhibition plus SEM of three independent experiments compared to the mock control (DMSO). A positive control (CAS 745028-76-6) was used at a concentration of 1 µM. (**B**) Chemical structures of MF-11, MF-14, and MF-15 with 2′-(2-hydroxy)-benzyl moiety are marked in red.

In a previous study, Mayr et al. described the general suitability of various natural chalcones as potential AKR1C3 inhibitors [39]. In this work, 11 closely related natural products with a dihydrochalcone or chalcone skeleton were submitted to enzyme inhibition assays on 17β-hydroxysteroid dehydrogenases 3 and 4, but most importantly AKR1C3. Three of those compounds, which were either isolated from the plant *Melodorum fruticosum* (MF-11 and MF-14) or synthetically derived from MF-14 (MF-15), proved to be the most effective in an AKR1C3 inhibition assay as described by Schuster et al. [40]. Briefly, the AKR1C3 enzyme was transformed into E. coli and the obtained lysate incubated with the test compounds, NADPH, and a radioactively labelled AKR1C3 substrate before being analyzed on HPLC coupled to online scintillation counting as described under material and methods. Substrate and product peaks were then chromatographically integrated and compared to the mock control. The described assay setup allows for a readout specific for AKR1C3 inhibition. At a concentration of 10 µM, MF-11, MF-14, and MF-15 turned out to be the most promising compounds with 47%, 62%, and 87% inhibition of AKR1C3 enzyme activity, respectively (see Figure 1). These three inhibitors were therefore selected for further cell culture experiments.

### 2.2. Amongst the Three Natural Compounds, MF-15 Displays the Most Potent Growth-Inhibitory Capacity in 22Rv1 Prostate Cancer Cells

**Figure 2:**
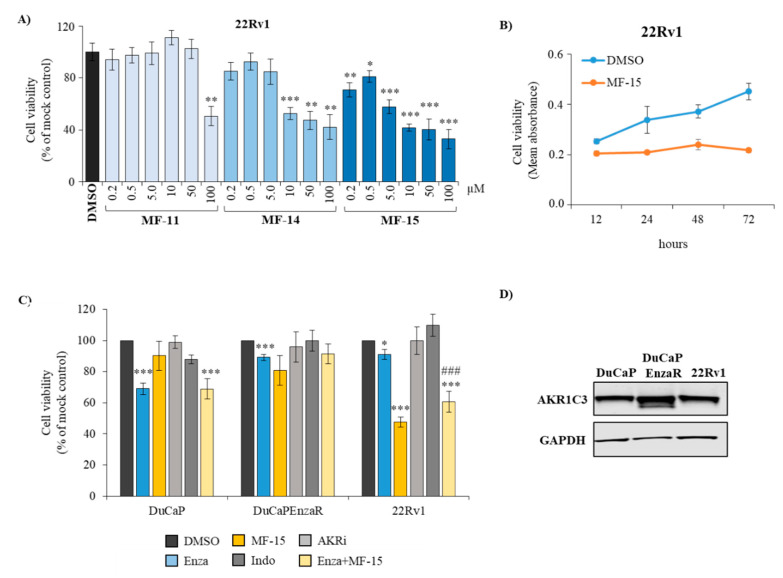
Anti-proliferative effects of AKR1C3 inhibitors in various prostate cancer cell lines. (**A**) 22Rv1 cells were treated with increasing concentrations of the natural chalcones MF-11, MF-14, and MF-15 dissolved in RPMI + 10% CS-FCS over 5 days or (**B**) with 10 µM MF-15 over various time points. (**C**) DuCaP, DuCaP EnzaR and 22Rv1 cells were seeded into 96-well plates and treated with MF-15 (10 µM), indomethacin (indo, 20 µM), AKRi (50 µM), enzalutamide (enza, 5 µM) or a combination of enzalutamide (5 µM) and MF-15 (10 µM) over 5 days as described under material and methods. Cell viability was measured through colorimetric MTS cell viability assay (Promega) and indicated as percentage of mock control (DMSO). Data represent the mean ±SEM from three independent experiments. Statistical comparisons to the mock control were expressed with an asterisk (* *p* < 0.05, ** *p* < 0.01, *** *p* < 0.001), comparisons to enzalutamide with a hash key (#). (**D**) Western blot analysis of AKR1C3 and glyceraldehyde 3-phosphate dehydrogenase (GAPDH) of DuCaP, DuCaP EnzaR, and 22Rv1 cells. Representative images of blots were taken with Image Studio software (Li-Cor).

To validate the anti-neoplastic effects of the compounds, we incubated 22Rv1 prostate cancer cells with increasing concentrations of MF-11, MF-14 and MF-15 over 5 days. As shown in Figure 2A, MF-11 did not inhibit cell viability at concentrations up to 50 µM. Only a concentration of 100 µM resulted in significant inhibition of 22Rv1. MF-14, on the other hand, already induced a significant growth retardation at a concentration of 10 µM. The most potent compound was MF-15, which significantly reduced cell viability at a concentration of 0.2 µM. At a concentration of 10 µM, MF-15 resulted in more than 50% growth inhibition compared to the mock control that was apparent as early as 48 h after drug addition (Figure 2B).

To further investigate cell line-dependent effects, we validated MF-15 in two other AR-positive prostate cancer cell lines. As shown in Figure 2C, 10 µM MF-15 significantly decreased cell viability of enzalutamide resistant DuCaP EnzaR cells but only weakly affected growth of parental DuCaP cells, which were sensitive to enzalutamide. Combined treatment of DuCaP cells with MF-15 and enzalutamide did not enhance the effect of enzalutamide alone. Still, the strongest effect of MF-15 was observed in 22Rv1 cells. In these cells, combined treatment with MF-15 and enzalutamide was more effective than enzalutamide alone (Figure 2C). Overall, these variances in response to MF-15 could not be explained by differences in AKR1C3 expression among the three cell lines as shown by Western blotting (Figure 2D). Notably, MF-15 was more effective than two other AKR1C3 inhibitors, indomethacin and AKRi, which were applied at concentrations of 20 µM and 50 µM, respectively (Figure 2C) and exhibited an inhibitory effect that was similar to the chemotherapeutic drug docetaxel (Figure S1A,B). In addition, MF-15 also significantly inhibited AR-negative PC-3 cells (Figure S1B).

### 2.3. MF-15 Inhibits 3D Spheroid Growth of Enzalutamide Resistant Du/CAF Co-Cultures

**Figure 3:**
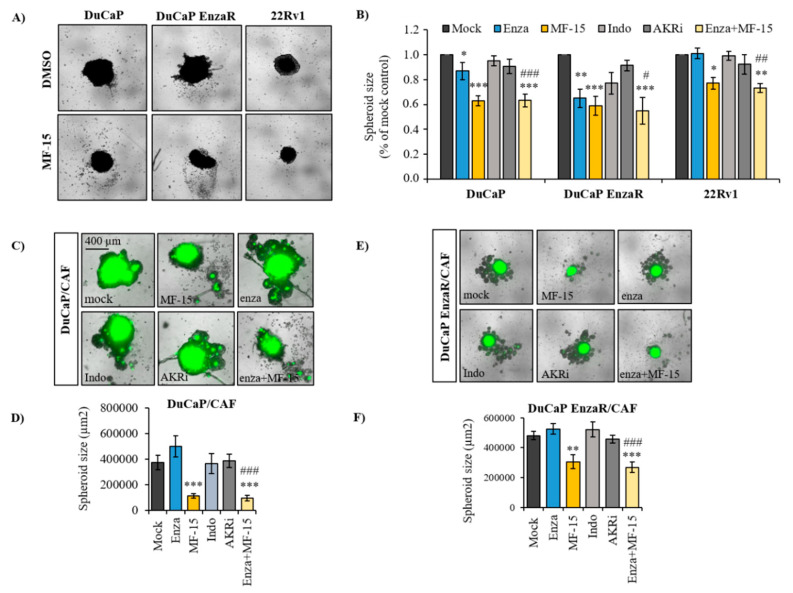
Anti-neoplastic effects of MF-15 in prostate cancer spheroids. (**A**) 22Rv1, DuCaP, and DuCaP EnzaR (8000 cells per well) or (**C**,**E**) DuCaP and DuCaP Enza R with GFP-labeled CAFs at a ratio of 1:1 (4000 + 4000 cells per well) were seeded into ULC 96-well plates (Corning) and allowed to form spheroids over 4 days. MF-15 (10 µM), indomethacin (indo, 20 µM), AKRi (50 µM), enzalutamide (5 µM) and a combination of enzalutamide (5 µM) with MF-15 (10 µM) were added at days 4 and 8 in RPMI + 10% CS-FCS. (**B**,**D**,**F**) Spheroid size was determined at day 10, and (**A**,**C**,**E**) representative images were taken with IncuCyte S3 software. Data represent the mean ±SEM from three independent experiments. Statistical comparisons to the mock control were expressed with an asterisk (* *p* < 0.05, ** *p* < 0.01, *** *p* < 0.001) and comparisons to enzalutamide with a hash key (#).

Previous findings by our group have demonstrated the usefulness of 3D tumor spheroids as relevant tissue-mimicking in vitro tools for drug testing [41]. Therefore, we further investigated the effect of MF-15 on prostate cancer spheroid growth. As summarized in Figure 3A, MF-15 significantly inhibited spheroid growth of all three cell lines at a concentration of 10 µM. By contrast to the effect of MF-15 in 2D culture, a significant anti-proliferative effect of MF-15 was also clearly seen in DuCaP spheroids. A less pronounced effect was observed in 22Rv1 spheroids. These differences in response to MF-15 in 3D spheroid culture may be due to variances in the spheroid size despite seeding equal cell numbers. In fact, 22Rv1 formed smaller spheroids than DuCaP and DuCaP EnzaR. Again, indomethacin and AKRi failed to significantly affect spheroid size. A combination of enzalutamide and MF-15 did not further increase the potent effect of MF-15 alone. Of notice, DuCaP EnzaR spheroids were inhibited by enzalutamide in this study, whereas 22Rv1 spheroids showed the expected weak response to the anti-androgen.

We next investigated MF-15 in co-culture spheroid assays. In a previous study, we demonstrated that prostate cancer cells become enzalutamide-resistant upon spheroid co-culture with CAFs, associated with increased production of pro-inflammatory cytokines and elevation of steroid biosynthesis with significant upregulation of AKR1C3 in particular [23]. Corresponding with our previous study, enzalutamide failed to affect DuCaP/CAF spheroid size whereas treatment with MF-15 resulted in a significant reduction of spheroid size compared to the mock control (Figure 3B). Similar results were obtained in DuCaP EnzaR/CAF spheroids (Figure 3C). Notably, enzalutamide was ineffective in both co-culture models. Similarly, indomethacin and AKRi were not able to affect co-culture spheroid growth. Results were confirmed by cell viability assays (Figure S1C,D).

### 2.4. MF-15 Inhibits Expression of AR Regulated Genes through Downregulation of AR Protein

**Figure 4:**
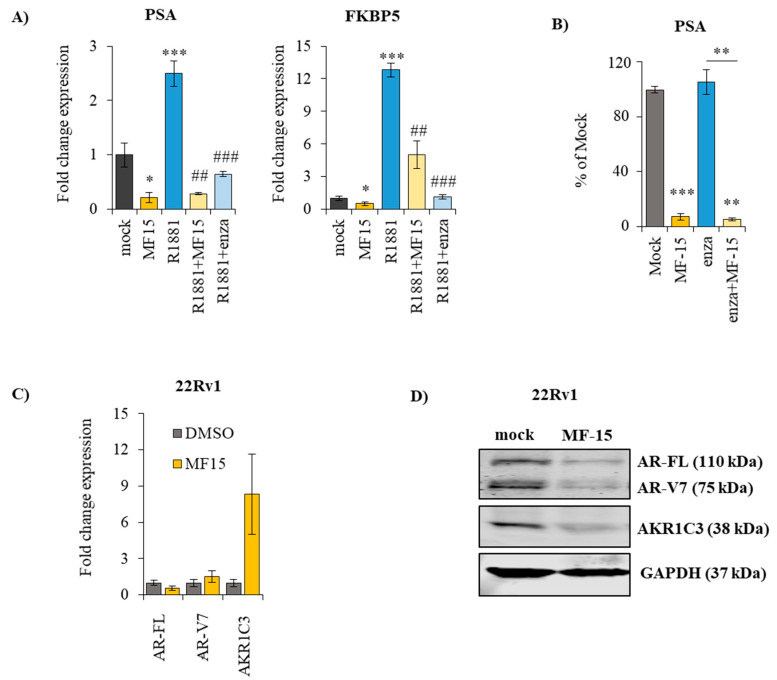
MF-15 reduces the expression of androgen-regulated genes, diminishes PSA production and decreases the expression of AR-FL and AR-V7. (**A**) Expression of the androgen-regulated genes PSA and FKBP5 was determined with real-time qPCR following treatment of 22Rv1 cells with the indicated drugs over 3 days. After normalization to the housekeeping gene HMBS, fold change expression was calculated to the mock control for each individual experiment. (**B**) PSA levels were measured in the cell culture supernatant after treatment of 22Rv1 cells with 10 µM MF-15 on a Cobas 8000 instrument (Roche) and calculated as percentage of mock control (DMSO). (**C**) Expression of AR-FL, AR-V7, and AKR1C3 was assessed by real-time qPCR in 22Rv1 cells treated with MF-15 or DMSO over 3 days. Expression of each gene was normalized to HMBS and expressed as fold change to the mock control. (**D**) Western blot analysis of AR-FL, AR-V7 and AKR1C3 was performed as described under material and methods. Glyceraldehyde 3-phosphate dehydrogenase (GAPDH) was used as loading control. Representative images of blots of three individual experiments were taken with Image Studio software (Li-Cor). Data represent the mean ±SEM from three independent experiments. (statistical comparison to mock control: * *p*< 0.05, ** *p* < 0.01, *** *p* < 0.001; statistical comparison to R1881: # *p*< 0.05, ## *p* < 0.01, ### *p* < 0.001).

Previous AKR1C3 inhibitors were shown to exert dual inhibitory effects on AKR1C3 and AR [22,24]. To explore a potential effect on AR signaling, we first determined the expression of androgen-regulated genes in 22Rv1 in response to MF-15. As revealed by qPCR, the expression of both PSA and FKBP5 was significantly diminished after treatment with 10 µM MF-15 in the absence and presence of the synthetic androgen R1881 (Figure 4A). These data were further corroborated by significantly reduced levels of PSA in the cell culture supernatant of 22Rv1 cells after treatment with MF-15 (Figure 4B), suggesting that MF-15 might in fact impair the AR itself.

To further investigate this, we next examined if MF-15 affected AR expression. As shown in Figure 4C, MF-15 only moderately reduced the expression of AR-FL and AR-V7. The expression of AKR1C3, on the other hand, was significantly upregulated in response to MF-15, suggesting a negative feedback mechanism. On the protein level, AR-FL and AR-V7 protein levels were significantly reduced by MF-15 compared to the mock control (Figure 4D), indicating that MF-15 inhibits the expression of androgen-regulated genes through downregulation of AR expression. Notably, on protein levels, also AKR1C3 expression was decreased by MF-15.

### 2.5. MF-15 Inhibits AR-FL and AR-V7 Reporter Gene Activity

**Figure 5:**
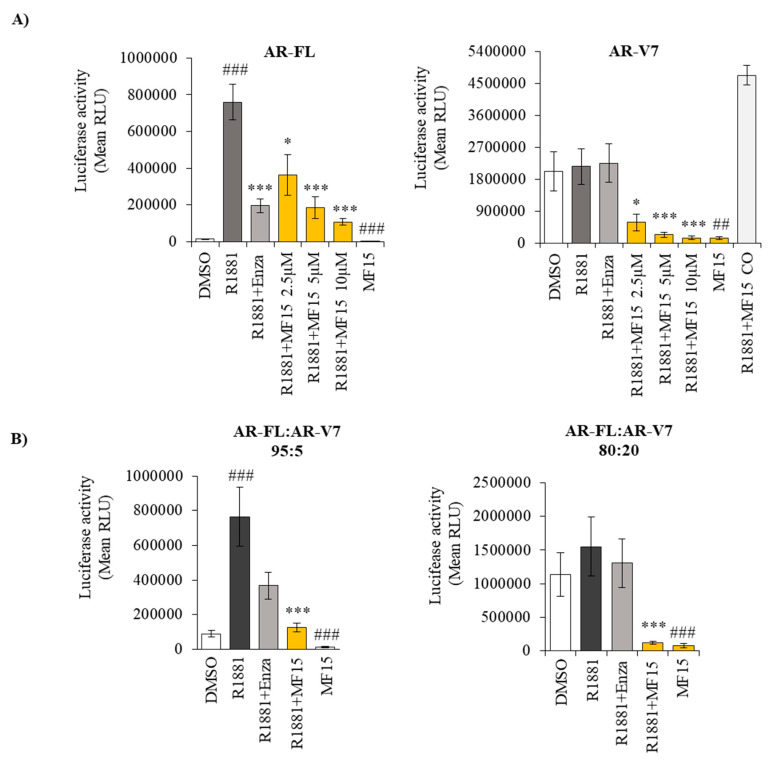
MF-15 inhibits AR-FL and AR-V7 reporter gene activity. (**A**) AR negative PC-3 cells were transiently transfected with full-length AR (AR-FL) or AR-V7 alone and seeded into 96-well plates in RPMI + 2% FCS supplemented with 10 µM MF-15 alone or with increasing concentrations of MF-15 or 5 µM enzalutamide (Enza) and 0.2 nM of the synthetic androgen R1881. To exclude unspecific interpolation of MF-15 with the assay, luciferase activity was measured following treatment of transfected cells with 0.2 nM R1881 and 10 µM MF-15 for 2 h (R1881+MF-15 CO). (**B**) PC-3 cells were transiently transfected with AR-FL and AR-V7 at different ratios (95:5 and 80:20) and treated with 10 µM MF-15 alone or with 10 µM MF-15 or 2.5 µM enzalutamide and 0.2 nM R1881. Luciferase reporter gene activity was determined with Nano Glow Dual Assay (Promega) by measuring absorbance on a CYTATION multiplate reader instrument. Data represent the mean ±SEM from three independent experiments. Statistical comparisons to the mock control were expressed in the graph with a hash key (## *p* < 0.01, ### *p* < 0.001) and comparisons to R1881 with an asterisk (*) (* *p*< 0.05, *** *p* < 0.001).

We next evaluated the influence of MF-15 in AR activity using a luciferase reporter gene assay. We used AR-negative PC-3 cells for these experiments to avoid potential confounding effects of endogenous AR and transiently transfected them with full-length AR (AR-FL). As shown in Figure 5A, MF-15 significantly inhibited R1881-induced AR-FL activity in a concentration-dependent manner. At a concentration of 10 µM, MF-15 was even more effective than enzalutamide, which was used at a concentration of 2.5 µM. Also in the absence of R1881, MF-15 was able to significantly reduce AR-FL activity. Most importantly, MF-15 also dose-dependently inhibited AR-V7 activity in PC-3 cells transiently expressing AR-V7 in the absence and presence of R1881 (Figure 5A). These strong effects of MF-15 were not caused by unspecific interpolation with the assay as MF-15 did not inhibit luciferase activity when administered 2 h before measurement. Of notice, basal activity was much higher with AR-V7 compared to AR-FL. In addition, enzalutamide did not affect AR-V7 due to the lack of the LBD.

Since AR-V7 transcript levels usually occur at substantially lower expression levels than AR-FL in vivo (42), we next transfected PC-3 cells with AR-FL and AR-V7 at two different ratios (95:5 and 80:20), thereby mimicking a more likely patient situation. As summarized in Figure 5B, enzalutamide lost its inhibitory activity with increasing amounts of AR-V7. Correspondingly, basal luciferase activity in the absence of R1881 increased with elevated AR-V7 levels. Independent of the amount of AR-V7, 10 µM MF-15 significantly inhibited luciferase activity in the absence and presence of R1881.

### 2.6. MF-15 Is a Non-Competitive AR Inhibitor with Probable Binding to the AR-DBD

**Figure 6:**
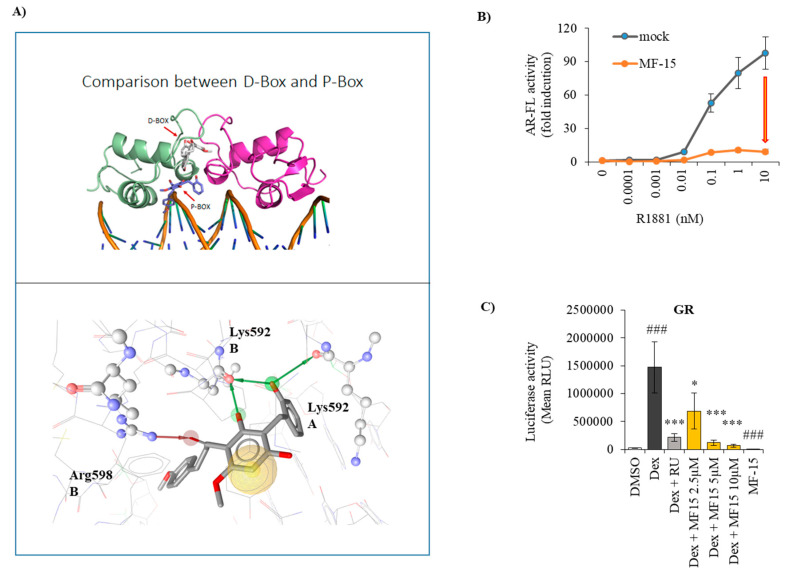
MF-15 targets within the AR-DBD. (**A**) Upper image: Docking poses of MF-15 in the D-box (white) and the P-box of the AR-DBD (blue). The A chain of AR is shown in green and the B chain in magenta. Lower image: Interactions of MF-15 within the P-box binding site. Green arrows signify hydrogen bond donor interactions with Lys592 (from the A and B chain), the red arrow marks a hydrogen bond acceptor interaction with Arg598. The yellow sphere represents hydrophobic contacts between ligand and binding pocket. (**B**) PC-3 cells were transiently transfected with AR-FL and incubated with increasing concentrations of R1881 without (blue curve) or with 10 µM MF-15 (orange curve) in the reporter gene assay described before. Data represent the mean ±SEM from three independent experiments. (**C**). PC-3 cells were transiently transfected with GR under the control of a ARE2TATA promoter and seeded into 96-well plates in RPMI + 2% FCS supplemented with 10 µM MF-15 alone or with increasing concentrations of MF-15 or 5 µM mifepristone (RU) and 1 nM of dexamethasone (Dex). Luciferase reporter gene activity was determined with Nano Glow Dual Assay (Promega) by measuring absorbance on a CYTATION multiplate reader instrument. Data represent the mean ±SEM from three independent experiments. Statistical comparisons to the mock control were expressed in the graph with a hash key (### *p* < 0.001) comparisons to R1881 with an asterisk (*) (* *p*< 0.05, *** *p* < 0.001).

Based on the fact that MF-15 strongly inhibits AR-V7, we hypothesized that the inhibitor most likely does not bind to the LBD of the AR as the anti-androgen enzalutamide does. To further elucidate the potential binding sites of MF-15 within the AR protein, we performed molecular docking studies on known binding sites aside from the AR-LDB. We first focused on the AR-DBD, where two binding sites, the P-box and the D-box amino acid motifs, have been previously described in the literature. The P-box is involved in binding to DNA, while the D-box mediates dimerization [13,36]. As demonstrated in Figure 6A, docking to the P-box resulted in a plausible binding mode for the molecule MF-15. We found a docking score of 35.98 and predicted MF-15 to form hydrogen bonds with Arg598 and Lys592 of the B-chain as well as with Lys592 of the A-chain (Figure 6A), suggesting that binding of MF-15 in this position of the AR-DBD would effectively block DNA binding. Docking in the D-box, on the other hand, resulted in steric clashes and extremely low docking scores (data not shown).

To further investigate the binding mode of MF-15 to the AR, we measured reporter gene activity after incubation of PC-3 cells transiently transfected with AR-FL with increasing concentrations of R1881 in the absence or presence of 10 µM MF-15. As shown in Figure 6B, MF-15 shifted the concentration response curve of R1881 towards the *x*-axis compared to the mock control, suggesting non-competitive inhibition of AR transcriptional activity. These data suggest that MF-15 acts differently from enzalutamide, which is known to be a competitive AR antagonist [9,42]. To further explore if MF-15 binds within the AR-DBD, we investigated if MF-15 cross-reacts with the DBD of the glucocorticoid receptor (GR), a related nuclear steroid receptor with high sequence similarity in its DBD (77–80%) [13,34,36]. As shown in Figure 6C, 10 µM MF-15 exhibited significant inhibition of the transiently expressed human GR. Furthermore, dexamethasone-stimulated GR activity was significantly and dose-dependently decreased by MF-15, suggesting that MF-15 most likely binds to the DBD.

## 3. Discussion

Resistances to potent anti-androgens like enzalutamide and abiraterone acetate are becoming a growing problem in the treatment of advanced prostate cancer. Therefore, the development of new drugs, which would help to overcome these resistances, is largely demanded. AKR1C3 has gained increasing attraction as a novel target based on studies demonstrating its impact on prostate tumor progression, mCRPC and development of resistances to enzalutamide and abiraterone acetate [14,23,24,43,44,45,46].

Several potent AKR1C3 inhibitors, including steroidal, non-steroidal and natural compounds, have been developed so far [1]. In the present study, we tested the antineoplastic effects of three chalcones in prostate cancer. They showed significant inhibitory activity on AKR1C3 in enzymatic assays with dihydrochalcone MF-15 as the most effective compound. This may be due to structural differences among the three compounds. In contrast to MF-11, MF-14 and MF-15 are 2′-(2-hydroxy)-benzylated. In addition, MF-15 is lacking the α-β double bond within the chalcone skeleton, thereby adding more flexibility to the overall structure of the protein. It therefore seems likely that for a favored protein binding of these compounds, both a 2′-(2-hydroxy)-benzyl moiety together with a rotable α-β single bond are beneficial.

In vitro cell culture experiments revealed that 10 µM MF-15 significantly inhibited various prostate cancer cell lines, including enzalutamide resistant DuCaP EnzaR and 22Rv1 cells, though with varying response rates. Western blot analysis, however, revealed substantial levels of AKR1C3 in all three cell lines, thereby suggesting that a low response rate to MF-15 can hardly be explained by faint AKR1C3 expression. In addition, we showed here that MF-15 also effectively prohibited 3D spheroid growth. Most importantly, MF-15 was also highly effective in inhibiting spheroid growth of DuCaP/CAF and DuCaP EnzaR co-culture spheroids, which were previously shown to exhibit increased AKR1C3 expression associated with enzalutamide resistance and a strong pro-inflammatory phenotype [23]. In comparison with MF-15, two purchased AKR1C3 inhibitors, indomethacin and AKRi, only moderately affected cell and spheroid growth even when applied at concentrations of 20 µM and 50 µM. Previous studies have shown that indomethacin alone did not inhibit 22Rv1 cells at concentrations up to 20 µM, but efficiently re-sensitized cells to enzalutamide in vitro and in vivo [14,24]. Indomethacin is a known AKR1C3 inhibitor, which also inhibits cyclooxygenases (COX-1, COX-2) and which is widely used as a nonsteroidal anti-inflammatory drug in the treatment of fever, pain or inflammation [29].

Another interesting aspect of some previously described AKR1C3 inhibitors is their bifunctional activity on AKR1C3 and the AR. The group of Verma et al. for instance reported on the antineoplastic activity of an AKR1C3 inhibitor called KV-37, which reduced prostate tumor cell growth in vitro and in vivo associated with a reduction of PSA expression and reduced AR transactivation [22]. In our study, MF-15 also significantly inhibited the expression of two androgen-regulated genes, FKBP5 and PSA, in enzalutamide-resistant 22Rv1 cells. In addition, we found that MF-15 significantly reduced PSA production and AR reporter gene activity associated with downregulation of AR protein, suggesting a direct impact on AR signaling. Even more important, MF-15 also inhibited AR-V7 reporter gene activity and induced a downregulation of AR-V7 expression in 22Rv1 cells. In numerous previous studies, the occurrence of the constitutively active AR-V7 splice variant lacking the C-terminal AR-LBD has been strongly linked to prostate tumor progression as well as to failures in the response to enzalutamide [15,42,47,48,49,50,51,52,53]. Furthermore, the presence of AR-V7 mRNA in circulating tumor cells of patients with metastatic CRPC has been shown to predict poor response to AR antagonists [15]. Drugs having the ability to suppress both AR-FL and AR-V7, and would therefore be of much better therapeutic efficacy, in particular in CRPC. MF-15 was shown to significantly and dose-dependently inhibit AR-FL and AR-V7, whereas enzalutamide—as expected—failed to inhibit reporter gene activity in the presence of AR-V7. Since patients, which are positive for AR-V7, usually show substantially low AR-V7 compared to relatively high AR-FL transcript levels at the same time [54,55,56], we also used various ratios of AR-FL:AR-V7 (95:5, 80:20) to mimic a more patient-like situation in our experimental settings. Of notice, not only was the basal reporter gene activity of AR-V7 much higher than that of AR-FL but, even more importantly, the inhibitory effect of enzalutamide decreased with increasing expression levels of AR-V7. Because MF-15 is also able to inhibit AR-V7 lacking the AR-LBD, it is highly considered that MF-15 acts via a different mechanism than enzalutamide, which is known as a competitive AR antagonist that binds within the AR-LBD [9,42]. In fact, further AR activity assays revealed that MF-15 non-competitively inhibits the AR. This may also be a possible reason why a combination of MF-15 and enzalutamide did not give a benefit over the effects of MF-15 alone. Additionally, it should be noted that we only tested MF-15 at a concentration of 10 µM, which was found to be highly effective when the drug was administered alone. Further experiments with much lower drug concentrations are warranted to clearly define a combination of MF-15 with AR-targeting agents.

Molecular docking simulations on known binding sites of the AR-DBD were conducted to propose a mechanism of action for MF-15 on the AR. The best results were found directly in the AR-DBD. These findings were further corroborated by reporter gene assays using the glucocorticoid receptor, which exhibits high similarity with the AR in its DBD [13,34,36]. MF-15 significantly inhibited GR activity in the absence and presence of dexamethasone, indicating that MF-15 binds to the DBD. Although a binding of MF-15 in the AR-NTD of course cannot be completely excluded, it should be further considered that the NTD of the AR represents an intrinsically disordered region, rendering the protein highly flexible, thereby allowing to form protein–protein interactions with multiple partners. In fact, more than 160 proteins including some inhibitors are known to interact with the AR-NTD [57]. Due to the high flexibility of this domain, however, it is unlikely that a non-covalent binder like MF-15 could induce a potent inhibitory effect on this part of the protein.

An AKR1C3 inhibitor like MF-15 with dual-inhibitory function on AKR1C3 and AR as well as on truncated AR variants holds the advantage of simultaneously inhibiting intratumoral androgen biosynthesis and the AR signaling cascade, thereby covering the action of antiandrogens such as enzalutamide and abiraterone acetate. Nevertheless, it should be noted that the growth-inhibitory effects of MF-15 were not restricted to AR-positive prostate cancer cells, since also AR-negative PC-3 cells were affected by MF-15 in our study, exerting a similar effect as the chemotherapeutic drug docetaxel. Anti-proliferative effects of ARK1C3 inhibitors in PC-3 cells have previously been shown by several other groups [58,59]. A potential mechanism was postulated by Sekine and coworkers, who showed that meclofenamic acid inhibited cell growth through attenuation of IGF-1-induced Akt activation [60].

Another advantage of an AKR1C3 inhibitor like MF-15 would be that a co-administration of prednisone, as it is common to prevent side effects of abiraterone acetate, would not be necessary [25,26]. MF-14 and other chalcones from *Melodorum fruticosum* have been shown to also possess significant anti-inflammatory properties, inhibiting microsomal prostaglandin E synthase-1 (mPGES-1), which represents a key enzyme in the synthesis of pro-inflammation prostaglandins [61]. Based on the knowledge that inflammation is correlated with tumor development and progression in general, a dual inhibition of mPGES-1 and AKR1C3 might represent a promising point in action [43,62]. Moreover, MF-15 had a strong effect in our co-culture spheroid models, which are characterized by a strong pro-inflammatory phenotype [23]. In summary, MF-15 is considered a novel and potent AKR1C3 inhibitor with additional AR targeting activity in the AR-DBD, thereby being potentially useful in the treatment of antiandrogen resistant CRPC.

From the clinical point of view, AKR1C3 inhibitors could be used in patients with enzalutamide resistance as a new additional treatment option, especially in cases where the patients do not meet the criteria for undergoing another mCRPC treatment like chemotherapy. However, clinical trials are warranted to proof the findings of this study.

## 4. Materials and Methods

### 4.1. Isolation of MF-11, MF-14 from Melodorum Fruticosum, and Preparation of MF-15

The leaves of *Melodorum fruticosum* were collected and extracted with CH_2_Cl_2_ as described in Engels et al. (61). The crude extracts (23 g) were subjected to silica gel column chromatography (424 g silica gel, Ø = 6.1 cm, L = 61 cm) and eluted with a gradient from petrol ether (100–0%), to ethyl acetate (0–100%), and methanol (0–100%), and collected time-dependently. The 170 obtained fractions were inspected with thin-layer chromatography using MF-14 as authentic standards and pooled to 10 comprehensive fractions (A-J). Fraction I (689 mg) was then separated over Sephadex^®^ LH-20 (Ø = 2 cm, L = 92 cm) using CH_2_Cl_2_:acetone (85:15, *v*/*v*) as a mobile phase to yield nine comprehensive fractions (I-a—I-i). Finally, 132.8 mg of fraction I-h were separated over Sephadex^®^ LH-20 (Ø = 1.5 cm, L = 70 cm) using methanol as a mobile phase to yield 35.1 mg MF-11 and 53.5 mg MF-14. MF-15 was obtained through catalytic hydration of MF-14. To this end, 23.8 mg MF-14 were dried and dissolved in methanol before the addition of Pd/C (Sigma Aldrich, Buchs, Switzerland) and H2 at room temperature under stirring. The reaction was monitored with thin-layer chromatography and stopped after 15 min by filtration. MF-15 was ultimately purified over Sephadex^®^ LH-20 with methanol as mobile phase and its identity and purity confirmed by ^1^H NMR by comparison literature (60). NMR spectra were acquired on a Bruker Advance II 600 NMR spectrometer (Bruker Biospin Rheinstetten, Germany). Finally, 18.5 mg MF-15 were obtained with a purity of >95%.

### 4.2. Docking Parameters for the AR

Docking simulations were carried out on a crystal structure of the AR-DBD (PDB entry 1r4i, Shaffer 2004) [63], using GOLD (version 5.2). Since there is no co-crystallized small molecule ligand in the crystal structure that could have been used for redocking, we aimed to recreate the docking pose of compound 25 from Li et al. [13] to optimize the docking workflow. DNA was deleted from the binding site and in the final settings the binding site was defined in a 9 Å radius around the coordinates 25.10, 81.08, 38.43. Goldscore was used as a scoring function.

### 4.3. Cell Lines

22Rv1 cells and PC-3 cells were obtained from the American Type Culture Collection (ATCC, Rockville, MD, USA) and cultured in RPMI (PAN-Biotech, Aidenbach, Germany) with 10% fetal calf serum (FCS, Gibco), 1% GlutaMAX^TM^ (Gibco) and 1% penicillin and streptomycin (Lonza). DuCaP cells were a gift from Prof. J. Schalken (Center for Molecular Life Science, Nijmegen, The Netherlands) and routinely cultured in RPMI with 10% FCS [36]. Enzalutamide-resistant DuCaP EnzaR cells have been previously established and characterized in our lab [38]. Immortalized CAFs have been previously established and described elsewhere [64] and were grown in RPMI with 10% FCS and 1% penicillin and streptomycin and 1x GlutaMAX™ (Gibco). CAFs were stably transfected with green fluorescent protein (GFP) by our group as described previously [41]. All cell lines were cultivated at 37 °C in a humidified atmosphere with 5% CO_2_. AR-V7 status of the used cell lines: 22Rv1: AR-V7 positive [37], DuCaP: AR-V7 positive [38], DuCaP EnzaR: AR-V7 positive [38], PC-3: AR-V7 negative [65].

### 4.4. Reagents

Enzalutamide (MedChemExpress), R1881 (Steraloids Inc. Newport RI), the AKR1C3 inhibitor (AKRi) (3-(4-trifluoromethyl)phenylamino) benzoic acid, Calbiochem), indomethacin (Sigma-Aldrich), dexamethasone (Selleck Chemicals), docetaxel (MedChemExpress) and mifepristone (Selleck Chemicals) were dissolved in dimethyl sulfoxide (DMSO). All solvents used for the isolation were purchased from VWR International (Darmstadt, Germany).

### 4.5. Cell Viability

Cell viability was determined via CellTiter 96^®^ Aqueous one solution cell proliferation assay (Promega). Briefly, 10 µL of reagent were added to 100 µL of cell culture medium, and absorbance was measured at 490 nm on a Cytation™ 5 Cell Imaging Multi-Mode Reader (BioTek). In each individual experiment, changes in cell viability were expressed as percentage of mock control.

### 4.6. Spheroid Culture

3D spheroids were established as described previously [23]. Briefly, 8000 cells were seeded into each well of a 96-well ULC ultralow attachment plate (Costar, 7007) and cultivated at 37 °C in a humidified atmosphere with 5% CO_2_. To obtain co-culture spheroids, DuCaP tumor epithelial cells were seeded together with CAFs at a ratio of 1:1 as previously described [41]. Spheroid size was determined with IncuCyte^®^ S3 LiveCell Analysis System.

### 4.7. Real Time Quantitative RT-PCR (qPCR)

Total RNA was isolated from cells with ExtractMe total RNA isolation Kit (Blirt, Gdansk, Poland) and quantified with the NanoDrop ND-2000c (Thermo Scientific). RNA was transcribed into cDNA by reverse transcription using SuperScript III reverse transcriptase (Invitrogen). qPCR was performed with TaqMan™ Assays (Thermo Fisher Scientific) for the quantification of FKBP5 (Hs01561006_m1), AKR1C3 (Hs00366267_m1), and hydroxymethylbilane synthase (HMBS, Hs00609297_m1), which was used as the endogenous control. Primer and probe sequences of AR-FL, AR-V7 and PSA were as follows: AR-FL (forward 5′-AGGATGCTCTACTTCGCCCC-3′, reverse 5′-ACTGGCTGTACATCCGGGAC-3′, probe 5′-FAM-TGGTTTTCAATGAGTACCGCATGCACA-TAMRA-3′), AR-V7 (forward 5′-CGGAAATGTTATGAAGCAGGGATGA-3′, reverse 5′-CTGGTCATTTTGAGATGCTTGCAAT-3′, probe 5′-FAM-GGAGAAAAATTCCGGGT-TAMRA-3′), PSA (forward 5′-GTCTGCGGC GGTGTTCTG-3′, reverse 5′-TGCCGACCCAGCAAGATC-3′, probe: 5′-FAM-CACAGCTGCCCACT GCAT CAGGA-TAMRA-3′). qPCR was carried out with ABI Prism 7500 Fast RT-PCR System (Applied Biosystems) cycler. Fold change in gene expression was determined using the mathematical model ratio 2^−ΔΔCT^ [66]. Values of genes of interest (GOI) were determined relative to HMBS. “Fold change” expression was calculated relative to the mock control (set 1) for each individual experiment.

### 4.8. Western Blotting

Cells were lysed using RIPA buffer supplemented with 0.5 mM PMSF, 2 µg/mL leupeptin, 2 µg/mL aprotinin, 2.5 mM NaF and 1% Triton X-100 by shaking at 4 °C for 1 h. Fifty micrograms of protein were separated using 4–12% NuPAGE Bis-Tris protein gels (Thermo Fisher ScientificTM, Vienna, Austria) and transferred onto a 0.2 µm nitrocellulose membrane (GE Healthcare, Vienna, Austria). Blocking of membranes and antibody incubation was performed in StartingBlockTM (PBS) blocking buffer (Thermo Fisher ScientificTM, Vienna, Austria) using the following antibodies: anti-AKR1C3 (clone NP6.G6.A6, 1:500, Sigma), anti-androgen receptor (1:500, Cell Signaling), anti-glyceraldehyde-3-phosphate dehydrogenase (GAPDH, 1:50000, Millipore). Visualization and quantification of protein bands were performed with Image Studio software Version 5.2 (LI-COR Biosciences).

### 4.9. Luciferase Reporter Gene Activity Assay

To assess reporter gene activity, AR negative PC-3 cells were transiently transfected with a CMV promoter-driven pFlag tagged full-length AR (AR-FL), the AR truncated splice variant 7 (AR-V7) or GR (pCMV6-XL5NR3C1, Origene), and a NanoLuc luciferase reporter gene under the control of a promoter element consisting of two consecutive canonical androgen response elements (AREs) and a TATA box (pNL1.1.-ARE2TATA) using ViaFect lipofection reagent (Promega). Cells were then seeded into 96-well plates in RPMI + 2% FCS at 37 °C overnight. Then, 0.2 nM R1881, 2.5 µM enzalutamide or 10 µM MF-15 were added. After 24 h, luciferase reporter gene activity was determined with Nano Glow Dual Assay (Promega) and measuring absorbance on a CYTATION multiplate reader instrument.

## 5. Conclusions

Our results show that the natural AKR1C3 inhibitor designated MF-15 achieves a significant inhibitory effect in enzymatic assays as well as anti-neoplastic effects in various prostate cancer cell cultures models. Moreover, MF-15 expressed a stronger inhibitory effect than other known AKR1C3 inhibitors under examination like indomethacin. Particularly interesting was the strong inhibitory effect of MF-15 on the AR and on its variant AR-V7 in cell lines, which were mostly insensitive to enzalutamide. Therefore, we are confident that a combination of AKR1C3 and AR inhibitory activity, as displayed by MF-15, is a powerful combination to combat prostate cancer. Especially the activity on AR-V7 could aid in overcoming the problem of enzalutamide resistance.

## Supplementary Materials

The following are available online at https://www.mdpi.com/2072-6694/12/8/2092/s1, Figure S1: Effects on the viability of 22Rv1 and AR-negative PC-3 cells and spheroid co-cultures.
