# Supplementary material for: Dual Inhibitory Action of a Novel AKR1C3 Inhibitor on Both Full-Length AR and the Variant AR-V7 in Enzalutamide Resistant Metastatic Castration Resistant Prostate Cancer
Source: Cancers (Basel). 2020 Jul 28;12(8):2092. doi: 10.3390/cancers12082092 (PMC7465893; doi:10.3390/cancers12082092)
Supplement: Supplementary file 1 [file cancers-12-02092-s001.pdf]

Supplementary Material: Dual Inhibitory Action of a Novel AKR1C3 Inhibitor on Both Full-Length AR and the Variant AR-V7 in Enzalutamide Resistant Metastatic Castration Resistant Prostate Cancer

Mona Kafka, Fabian Mayr, Veronika Temml, Gabriele Möller, Jerzy Adamski, Julia Höfer, Stefan Schwaiger, Isabel Heidegger, Barbara Matuszczak, Daniela Schuster, Helmut Klocker, Jasmin Bektic, Hermann Stuppner and Iris E. Eder

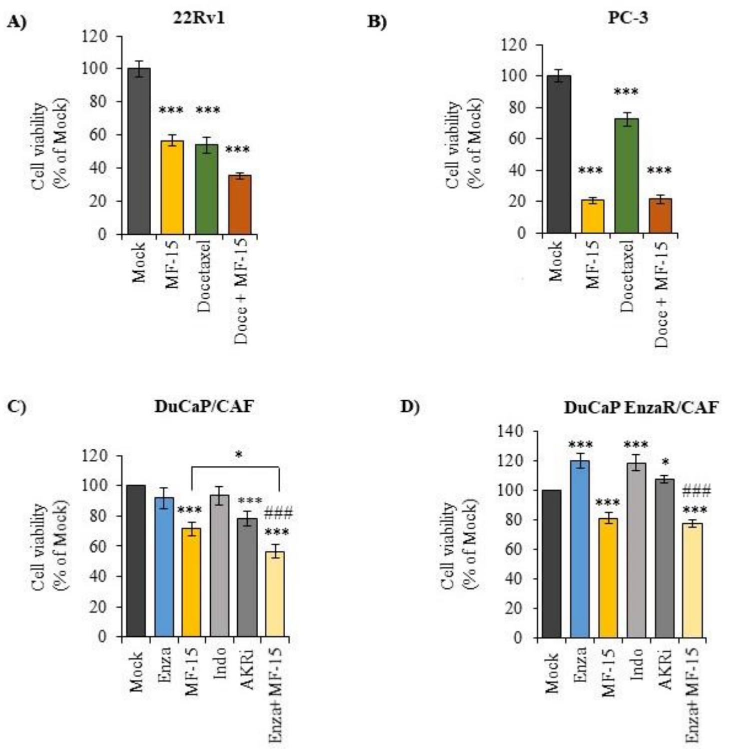

Figure 1. Effects on the viability of 22Rv1 and AR-negative PC-3 cells and spheroid co-cultures. 22Rv1 (**A**) and PC-3 (**B**) cells were seeded into 96 well plates and treated with MF-15 (10 µM), 12nM docetaxel, or a combination of the two drugs over 5 days as described under material and methods. Cell viability was measured through colorimetric MTS cell viability assay (Promega) and indicated as percentage of mock control (DMSO). Co-culture spheroids (**C**, DuCaP/CAF, **D**, DuCaP EnzaR/CAF) were established by seeding equal numbers of tumor cells and CAFs into ULC 96 well plates (Corning). MF-15 (10 µM), indomethacin (indo, 20 µM), AKRi (50 µM), enzalutamide (5µM) and a combination of enzalutamide (5 µM) with MF-15 (10 µM) were added at days 4 and 8 in RPMI + 10% CS-FCS. Cell viability was assessed by colorimetric MTS assay (Promega) and expressed as percentage of mock control. Data represent the mean ±SEM from three independent experiments. Statistical comparisons to the mock control were expressed with an asterisk (* $p < 0.05$, ** $p < 0.01$, *** $p < 0.001$), comparisons to enzalutamide with a hash key (### $p < 0.001$).

© 2020 by the authors. Licensee MDPI, Basel, Switzerland. This article is an open access article distributed under the terms and conditions of the Creative Commons Attribution (CC BY) license (http://creativecommons.org/licenses/by/4.0/).
